# Tools for using the International Rice Genebank to breed for climate-resilient varieties

**DOI:** 10.1371/journal.pbio.3002215

**Published:** 2023-07-06

**Authors:** Kenneth L. McNally, Amelia Henry

**Affiliations:** Rice Breeding Innovations Department, International Rice Research Institute, Los Baños, Laguna, Philippines; University of California, Davis, UNITED STATES

## Abstract

Of all crop species, rice has the most genetic potential for adaptation to climate change. Genebank accessions have been critical in developing improved stress-tolerant rice varieties. This Community Page highlights new tools and resources from the International Rice Research Institute for accelerating the identification and deployment of genes conferring climate change resilience.

## Teaser

Traditional rice varieties have been critical for developing improved stress-tolerant rice varieties. Tools to analyze the genome sequences of traditional varieties are accelerating the identification and deployment of genes conferring climate change resilience.

**Fig 1:**
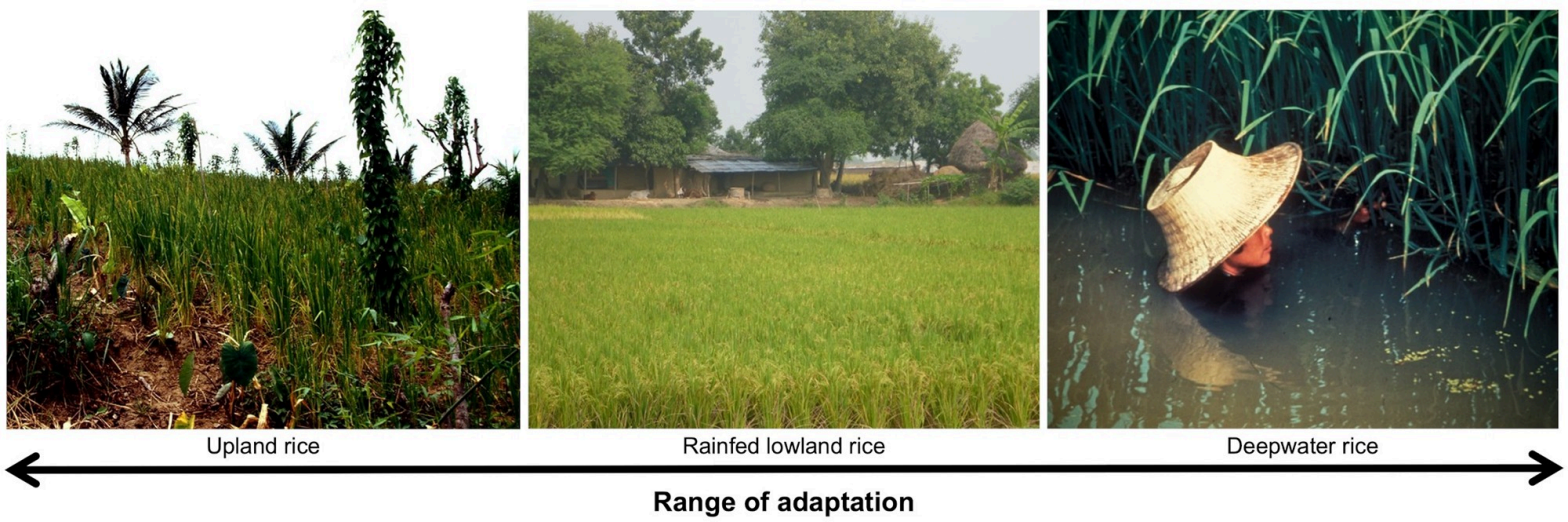
Traditional rice varieties are adapted to a wide range of climate conditions. An illustration of the range of stress-prone agro-ecosystems to which traditional rice varieties are adapted: upland (left), comprising aerobic soils that are often drought prone, nutrient deficient, and low input; rainfed lowland (center), comprising nonirrigated systems that are prone to drought, submergence, and salinity; and deepwater (right), in which water levels continuously rise during the growing season, often necessitating harvesting by boat. This large range of environmental adaptation is unique to rice, and these traditional varieties, which are maintained by Genebanks, serve as important genetic resources in breeding for climate change.

Climate change is associated with crop exposure to unpredictable temperatures, water levels, salinity, and elevated carbon dioxide. Due to its wide range of adaptability [1], of all crops, rice has the most genetic potential for adaptation to climate change, with traditional varieties adapted to conditions ranging from completely aerobic soil throughout the growing season (upland rice), through fluctuation from submerged to dry conditions within a single season (rainfed rice), to flooding with >5 m water (deepwater rice) (Fig 1). Improving rice adaptation to climate change–related stress is a major effort of the International Rice Research Institute (IRRI) and a key mandate directing our work to ensure rice food security. One important aspect of this work is Genebanks, seed repositories that conserve traditional varieties, improved varieties, and crop wild relatives. The IRRI Genebank maintains more than 130,000 types of cultivated rice and wild species with passport data (collection location and related information) available at GrinGlobal, enabling their potential effective use in breeding.

## Tools for using the IRRI Genebank

**Fig 2:**
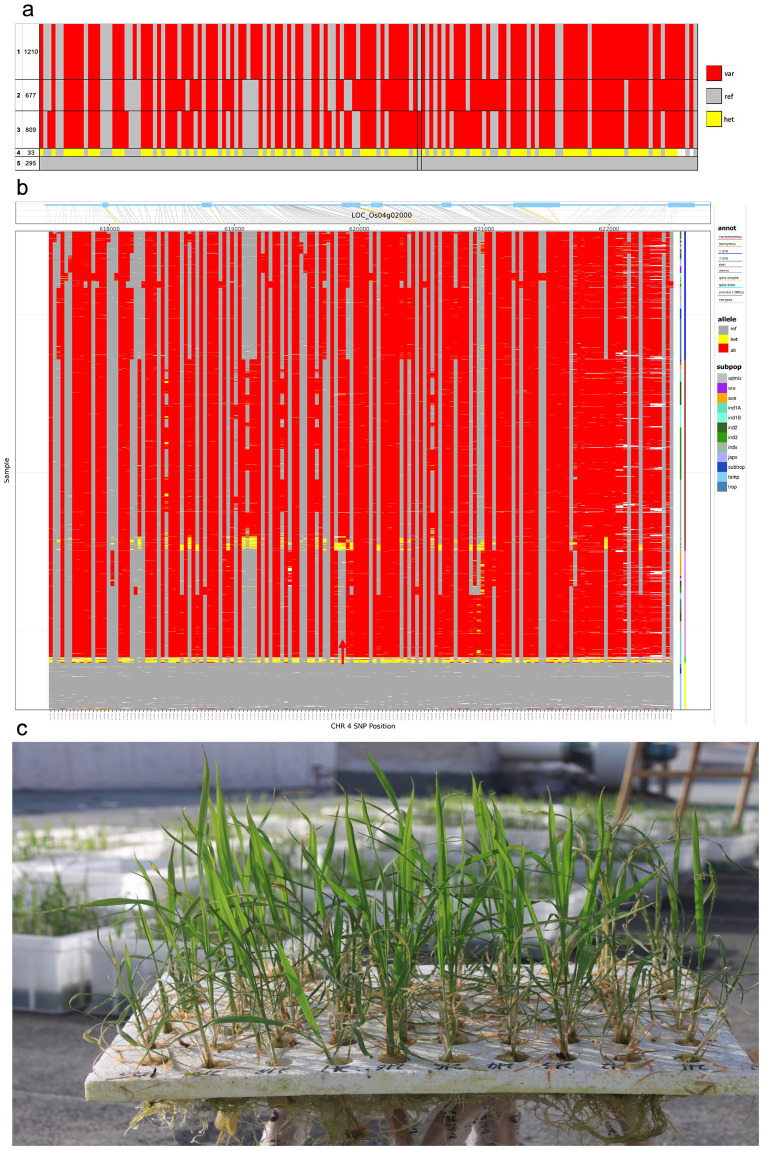
Using SNP-Seek to identify haplotypes for climate adaptation–related genes. Single nucleotide polymorphism (SNP) haplotypes for OsSTL1 (LOC_Os04g02000; a gene identified by Yuan and colleagues [5] to improve rice salt tolerance) in the 3K RG using SNP-Seek. **(a)** Across the 3K RG, 162 SNPs were found in the 4.8 M filtered SNP set that group into 3 major and 2 minor haplotypes. **(b)** Haplotype patterns across the 3K RG. Red arrow indicates position 619903 on Chr 4 where a nonsynonymous CDS SNP occurs. **(c)** Screening of germplasm for seedling stage salinity tolerance by Yuan and colleagues (image kindly provided by Prof. Zichao Li, China Agricultural University, Beijing, China). Breeding lines with improved salinity tolerance could be identified by screening for the haplotype carrying the favorable allele.

Traditional varieties have served as critical sources of stress tolerance that can be deployed through crop breeding to improve varieties that are stress sensitive but high yielding under favorable conditions. Examples of such “upgrading” of existing varieties to improve their adaptation to changing climate conditions include sub1 varieties (for submergence tolerance) and DRR 42 (for drought tolerance) [2]. Important resources enabling exploitation of traditional varieties are the genetic stocks (which have been purified from the traditional variety by single seed descent to ensure they are not mixed with other varieties), their genomic data, and tools to perform genetic analysis to identify potential stress-tolerant donors and favorable haplotypes. An important set of genetic stocks used extensively by the global community is the diverse set of 3,000 rice genomes (3K RG) originating from 89 countries, all of which have been sequenced [3]. This detailed 3K RG analysis has had 89k accesses and 699 Web of Science citations (Nature metrics as of 2023 June 20), indicating the work is of broad interest. The SNP-Seek database [4] provides access to the 3K RG sequence data and provides tools facilitating analysis for gene discovery of climate change–related traits, nutrition, yield, and other traits. Since 2014, SNP-Seek has served 212,581 sessions from 163 countries, with an average of 50 sessions per day (via Google Analytics). Through SNP-Seek, users can further explore identified genomic regions for given traits to help pinpoint genes and markers that can be used in breeding, for example, by obtaining variant matrices, exploring annotations of regions (including alignments and variants), constructing haplotypes that define the patterns, and supporting candidate genes in peaks after association analysis by adding evidence. Fig 2 illustrates use of SNP-Seek for identifying variants and creating haplotypes for genes of interest.

Another tool enabling analyses of single nucleotide polymorphism (SNP) variants is Crop Galaxy. Using Crop Galaxy and SNP-Seek variants, clients without access to advanced computing environments can accomplish genome-wide association studies (GWAS) and conduct post-GWAS analyses via the Galaxy web-based platform without installing tools on their local systems. This may be of particular interest to scientists in regions with limited computing infrastructure.

## Using the tools for trait discovery

These tools are already being used to discover traits important for adaptation to climate change, such as tolerance to salinity, drought, heat, and cold. Some examples using the diversity panels from the 3K RG for GWAS and candidate gene discovery follow.

Coastal areas are expected to be impacted by increased salinity due to rising sea levels. Kaur and colleagues [6] undertook analyses of the rice homologue of the Arabidopsis *MIZ1* gene, *Loc_Os02g47980*, which is involved in water sensing. Increased *OsMIZ1* expression was related to salinity and drought responses in lines from the 3K RG. In another study [5], GWAS and SNP analysis for seedling stage salinity tolerance was used to identify 2 novel candidate loci for salinity tolerance, *OsSTL1* (*LOC_Os04g02000*) and *OsSTL2*. OsSTL1 was similar to Arabidopsis stress-responsive protein 1 (SRP1); a nonsynonymous mutation in the coding sequence altered the predicted protein and was present in the superior haplotype (from *japonica* types) tolerant to seedling stage salinity (Fig 2). In addition to these examples of using SNP-Seek tools, gene annotations that describe function can be deeply explored to examine structural variants, providing more evidence for involvement in physiological, developmental, or regulatory processes.

Increased temperatures during critical growth phases can reduce yield; therefore, identifying regions involved in heat tolerance is necessary to improve rice. Yang and colleagues [7] performed GWAS for heat tolerance in 3K RG lines. Combining evidence from GWAS and expression analysis pinpointed 11 candidate genes. *LOC_Os05g07050* encodes a putative pre-mRNA processing splicing factor that controls removal of intronic RNA before translation. A mutation in tolerant types (where Leu replaced Ile) was found in this gene relative to the variety Nipponbare, which is a temperate *japonica* line and has been the gold standard rice genome since 2005. Changes in splice forms with exon replacements in expressed genes were seen under high temperature, indicating regulation of alternative spliced forms under high temperatures.

Cold is another stress affecting rice production that can occur during any growth stage; in fact, it is now possible to grow rice in some previously cold-affected regions due to climate change. Li and colleagues [8] identified *CTB2* as a candidate gene associated with cold adaptation by mediation of sterol metabolism in 3K RG lines with haplotypes for improved cold tolerance found in *japonica* types. Overall, the frequency of haplotypes in lines being developed by breeders will guide their use in breeding for climate change by prioritizing line advancement of favorable haplotypes or by crossing new alleles into advanced lines when the favorable haplotype is absent from the breeding pool.

## Future directions

Concurrent with climate change, there is an increasing global demand for rice that necessitates that rice breeding must target improved stress tolerance as well as genetic gain, i.e., consistently increasing yield. To this end, IRRI has implemented the new OneRice breeding strategy that involves stage gates to develop varieties adapted to climate change–related stresses in a given region, rather than upgrading individual varieties for tolerance to a single stress [9]. It implements elite × elite crossing, with novel alleles added for needed traits. The OneRice Trait Development pipeline for prebreeding links the Genebank to the elite breeding pool by providing guidelines for donor identification, gene/quantitative trait locus (QTL) introgression, validation, and development of “elite donor” lines that can be used in elite × elite crossing [10]. The Genebank tools described here are critical for moving promising traits/genes/QTLs forward in the Trait Development Pipeline.

Going beyond the 3K RG, we envision that the decreasing trend in sequencing costs will enable sequencing of all 130,000 accessions in the IRRI Genebank collection in the near future, creating what would be the Digital Rice Genebank (DRB). A matter of global concern is dematerialization of in-trust germplasm since digital sequence information (DSI) can guide genome editing, bypassing the need for seed, and thus disrupt the access and benefit sharing aspects embodied by the Nagoya Protocol of the Convention on Biological Diversity (CBD) and the International Treaty for Plant Genetic Resources in Food and Agriculture (ITPGRFA) [11]. Policy discussions among parties of the CBD and ITPGRFA, CGIAR scientists, and other concerned plant scientists (as expressed by the Global Plant Council DSI working group) must be resolved, respecting farmers’ rights, and ensuring that seed and DSI remain global public goods to be used for the benefit of humanity.

The DRB is being initiated by sequencing 10,000 additional Genebank accessions in collaboration with Prof. Rod Wing (KAUST and University of Arizona) and colleagues. These sequences, combined with 3K RG data and analyzed with high-quality reference genomes [12], will further enable improvements in rice to mitigate climate change–related stress. Creating multiple omics datasets (e.g., transcriptome, proteome, and metabolome) on selected subsets followed by integration/annotation of these data in SNP-Seek will improve the identification of genes involved in climate change.

In addition to improving the available resources to mine valuable climate change–related alleles from traditional rice varieties, a stronger linkage between Genebank-related work and current breeding objectives is needed. To this end, inclusion of more sequence information on released varieties and breeding lines in SNP-Seek is underway. Combined analysis of varieties and diverse traditional varieties promises to pinpoint the best sources and haplotypes for inclusion in breeding programs targeting stress improvement for climate change traits.

## Abbreviations

3K RG: 3,000 rice genomes
CBD: Convention on Biological Diversity
DRB: Digital Rice Genebank
DSI: digital sequence information
GWAS: genome-wide association study
IRRI: International Rice Research Institute
ITPGRFA: International Treaty for Plant Genetic Resources in Food and Agriculture
QTL: quantitative trait locus
SNP: single nucleotide polymorphism
SRP1: stress-responsive protein 1
